# Concurrent glucocorticoid taper enables reintroduction of alectinib after drug-induced rash

**DOI:** 10.1016/j.jdcr.2026.02.009

**Published:** 2026-02-12

**Authors:** Amy Liao, Ece Cali Daylan, Saiama Waqar, Jeffrey Ward, Spencer Ng

**Affiliations:** a Division of Dermatology, Department of Medicine, Washington University School of Medicine, St. Louis, Missouri; b Division of Oncology, Department of Medicine, Washington University School of Medicine, St. Louis, Missouri; c Department of Pathology and Immunology, Washington University School of Medicine, St. Louis, Missouri

**Keywords:** alectinib, ALK-positive non–small cell lung cancer, cutaneous adverse reaction, drug-induced rash, glucocorticoid taper

## Introduction

With the rise of precision medicine in oncology, targeted therapies are increasingly used to treat cancers with actionable mutations. Alectinib, a selective anaplastic lymphoma kinase (ALK) inhibitor, is currently approved as a first-line therapy option for patients with metastatic *ALK-*rearranged non–small cell lung cancer.^1^^,^^2^ Alectinib–associated cutaneous eruption, most commonly described as a morbilliform or macular and papular erythematous rash, is a known adverse effect of alectinib. Although the majority of rashes are classified as grade 1 to 2 according to the National Cancer Institute Common Terminology Criteria for Adverse Events, rare grade ≥ 3 reactions have been reported and may require treatment interruption or discontinuation. However, standardized guidelines for reintroduction after rash onset are lacking. Most published case reports describe restarting alectinib at very low doses followed by slow uptitration, with few patients tolerating a return close to the full standard dose of 600 mg twice daily.^3^, ^4^, ^5^ Our case series highlights an alternative approach: using a high-dose oral glucocorticoid taper alongside reintroduction of alectinib at a moderately reduced dose, without the need for slow desensitization.

### Case 1

**Fig 1:**
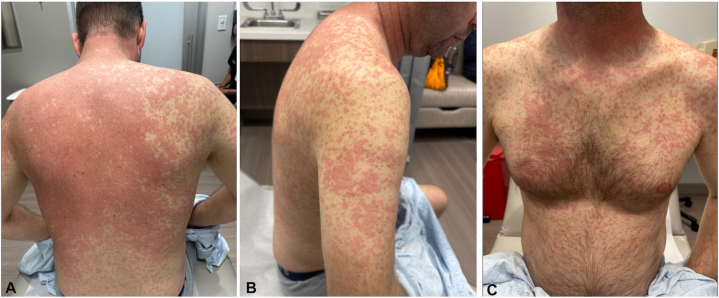
Pink papules coalescing into patches and plaques with a background of generalized erythema on the back **(A),** arms **(B),** and chest and abdomen **(C)** in case 1.

**Fig 2:**
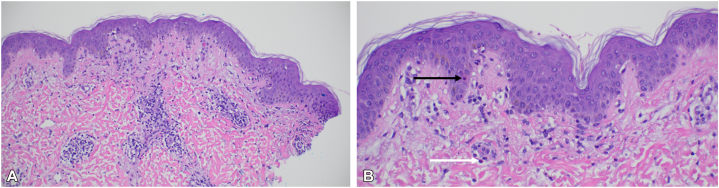
Punch biopsy showing focal interface dermatitis with a superficial perivascular mixed inflammatory cell infiltrate by hematoxylin and eosin staining (H&E; 20× magnification) **(A)**. Apoptotic keratinocytes (*black arrow*) and rare eosinophils (*white arrow*) **(B)**. (**A** and **B,** Hematoxylin-eosin stain; original magnifications: **A,** ×20; **B,** ×40.)

**Fig 3:**
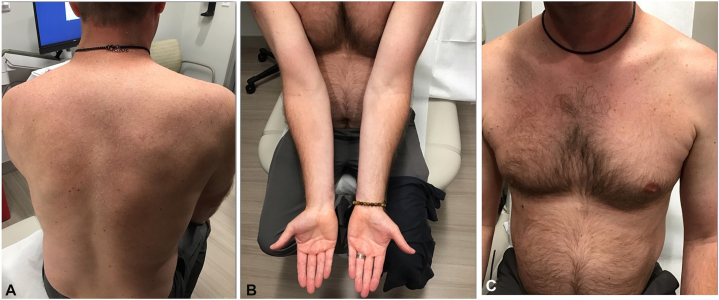
Improvement of rash on the back **(A),** arms **(B),** and chest **(C)** 2 weeks after initiation of prednisone taper in case 1.

A 44-year-old man with metastatic stage IVA lung adenocarcinoma had previously received one cycle of pembrolizumab and messenger RNA-4359 immunotherapy. Prior molecular testing, including fluorescence in situ hybridization, GatewaySeq next-generation sequencing, and peripheral circulating tumor DNA, revealed no clinically significant genomic variants. Given his never-smoker status, expanded genomic profiling with the Tempus xT 648-gene panel was subsequently pursued, and an *EML4::ALK* fusion was identified. Therefore, 1 month after his first and only cycle of immunotherapy, he was initiated on alectinib 600 mg twice daily. Twelve days after starting alectinib, he experienced a diffuse, pruritic grade 3 rash (graded per National Cancer Institute Common Terminology Criteria for Adverse Events) described as pink papules coalescing into plaques on a background of generalized erythema (Fig 1). He also presented with ear swelling, sore throat, ocular pruritus, and elevated liver enzymes (aspartate aminotransferase; AST 79 U/L and alanine aminotransferase; ALT 97 U/L). Although there was initial concern for drug reaction with eosinophilia and systemic symptoms, the latency from drug initiation was only 12 days, shorter than the typical 2 to 6 week onset, and the absence of eosinophilia or atypical lymphocytosis made the clinical presentation more consistent with an acute drug eruption. Skin biopsy showed focal interface dermatitis with eosinophils suggestive of drug reaction (Fig 2). Alectinib was held, and the patient received intravenous dexamethasone followed by a high-dose oral prednisone taper for 6 weeks, starting at 80 mg for 7 days and decreasing by 10 mg weekly. Over the next 2 weeks, his liver enzymes normalized, and his rash improved to mild, ill-defined erythema with superficial desquamation, without pruritus (Fig 3). Alectinib was reintroduced at 300 mg twice daily while continuing the steroid taper, now at 70 mg, with ongoing improvement of the rash. Over the next 3 weeks, the alectinib was gradually uptitrated to 450 mg twice daily, and the patient completed the prednisone taper without further recurrence of the rash. The dose was maintained at 450 mg due to persistent nausea at 600 mg. The patient has remained on 450 mg twice daily for a total of 7 months without interruption since resumption. At the time of manuscript submission, restaging CT of the chest, abdomen, and pelvis demonstrated an ongoing partial response to treatment, and he will continue on alectinib.

### Case 2

**Fig 4:**
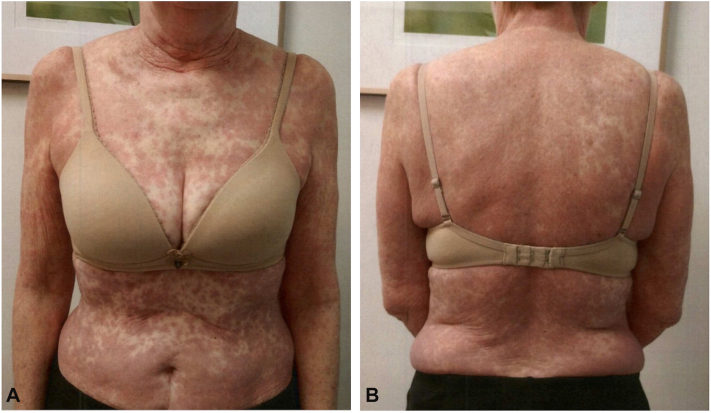
Erythematous blanching macules and papules on the chest, abdomen, bilateral upper extremities **(A)** and back **(B)** in case 2.

**Fig 5:**
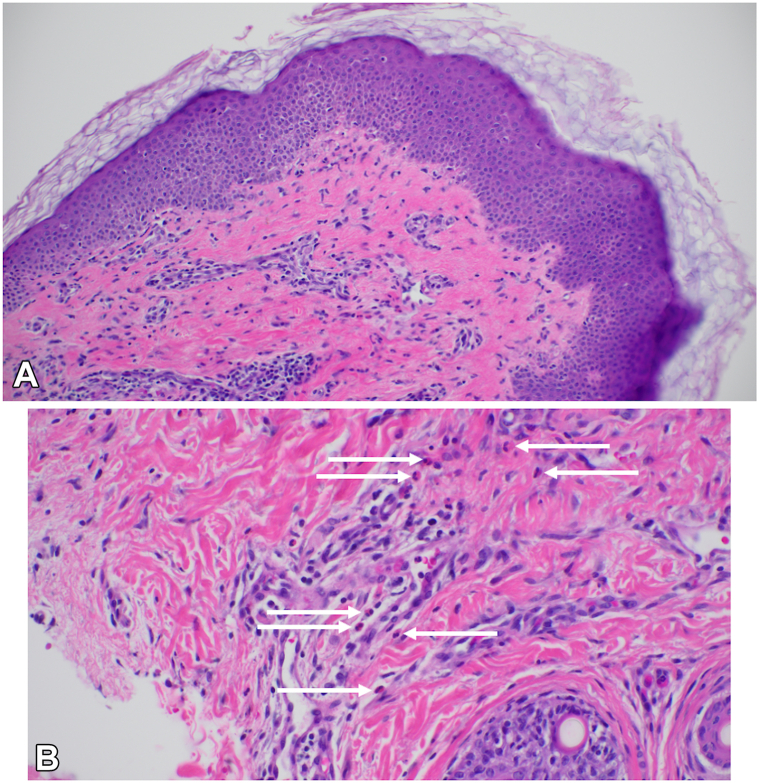
Punch biopsy showing superficial and deep perivascular and interstitial mixed inflammatory cell infiltrate containing lymphocytes, histiocytes, and eosinophils **(A)**. Mild features of spongiotic and interface dermatitis are also seen. The perivascular inflammatory infiltrate is rich with eosinophils (*white arrows*) **(B)**. (**A** and **B,** Hematoxylin-eosin stain; original magnifications: **A,** ×40; **B,** ×60.)

A 65-year-old woman with metastatic stage IVA non–small cell lung adenocarcinoma that harbored an *EML4::ALK* fusion gene was started on alectinib 600 mg twice daily. Ten days later, she experienced a sudden, diffuse, mildly pruritic grade 3 rash with fever. Examination showed widespread, blanchable erythematous macules and patches (Fig 4), glossitis, lip erosions, and mild cervical lymphadenopathy. Liver function tests, including AST, ALT, and alkaline phosphatase, remained within normal limits. Evaluation using the RegiSCAR criteria raised consideration of drug reaction with eosinophilia and systemic symptoms due to the presence of an eruptive macular rash, fever, lymphadenopathy (potentially attributable to underlying malignancy), edema, glossitis, and mucosal involvement. However, drug reaction with eosinophilia and systemic symptoms was considered less likely given the rapid onset of symptoms 10 days after initiation of alectinib and the absence of hepatic involvement. Overall, the clinical presentation was more consistent with alectinib-induced drug eruption. Skin biopsy demonstrated interstitial dermatitis with eosinophils, mild spongiosis, and interface changes, consistent with a drug-induced dermal hypersensitivity reaction (Fig 5). Alectinib was stopped, and prednisone 60 mg daily was initiated with a gradual taper. After 1 week, the patient underwent a 7-step rapid desensitization protocol for alectinib administered over 3 hours (total of 641 mg with step-wise doses of 1, 10, 30, 50, 100, 150, and 300 mg) and resumed 600 mg daily while tapering steroids, now at 40 mg. However, the rash recurred after the first dose, prompting a reduction to 150 mg daily. Over 2 weeks, the dose was titrated to 450 mg daily, and prednisone was tapered down to 15 mg, with improvement of the rash to only residual patchy erythema. One month later, she was escalated to a dose of 750 mg daily (divided between morning and evening) and completed the prednisone taper, achieving complete rash resolution without recurrence. The patient remained on this dose of alectinib for approximately 7 months, with a brief 2-week interruption due to mild transaminitis and shortness of breath concerning alectinib-associated pneumonitis, both of which resolved without complications. During therapy, she achieved a partial response with periods of stable disease. Treatment was ultimately discontinued because of mediastinal disease progression, prompting palliative radiation and transition to lorlatinib therapy.

### Case 3

**Fig 6:**
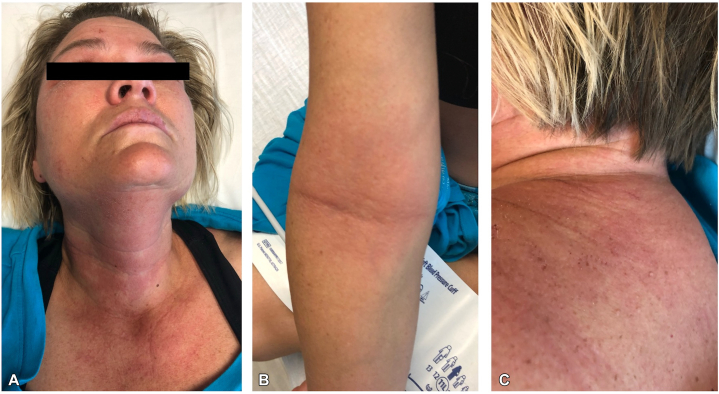
Facial edema with erythema and desquamation of the cheeks, neck, and upper chest **(A)**. Eczematous, edematous papules coalescing into plaques on the antecubital fossa **(B)** and upper back **(C)** in case 3.

A 49-year-old woman with metastatic stage IV right lung adenocarcinoma that harbored an *EML4::ALK* fusion gene was started on alectinib 600 mg twice daily, achieving significant improvement in disease burden per imaging. However, 78 days after initiation of alectinib, she experienced a new grade 2 rash and grade 2 pneumonitis. The patient was afebrile. Examination revealed facial swelling with erythema and diffuse, eczematous, edematous papules coalescing into plaques (Fig 6). Liver function tests (AST, ALT, and alkaline phosphatase) were within normal limits. Skin biopsy from the upper back showed spongiosis, focal subtle interface change, and a superficial perivascular and interstitial mixed inflammatory cell infiltrate with lymphocytes, histiocytes, and many eosinophils, consistent with a drug eruption. Alectinib was held, and a prednisone taper was started at 40 mg. After 7 days, with the rash improving, steroids were tapered down to 20 mg, and alectinib was resumed at 450 mg twice daily, without any titration. This dose of alectinib was maintained with complete rash resolution and no recurrence. Treatment was stopped 3 weeks later due to disease progression. Overall, the patient had been on alectinib for approximately 4 months.

## Discussion

Morbilliform or macular and papular erythematous rash is an infrequent but clinically significant adverse event associated with alectinib therapy. In the global randomized phase III ALEX trial, 13.9% of patients experienced a rash, with grade ≥ 3 severity observed in 2.0%.^6^ Similarly, the Japanese J-ALEX trial used a lower dose of alectinib at 300 mg twice daily, but still reported a rash incidence of 27.1%, with 2.9% of patients experiencing grade ≥ 3 rash.^7^ In both studies, rash was managed with dose interruption or reduction, without a standardized approach specific to cutaneous reactions. To date, there is no consensus on the optimal management of alectinib-associated rash. Only a limited number of case reports have described individual desensitization protocols, underscoring the need for more clinical guidance in managing this adverse effect.

Currently, most protocols describe slow desensitization strategies that begin at very low doses (eg, 20, 37.5, or 40 mg twice daily) followed by gradual uptitration over several weeks.^3^, ^4^, ^5^

Seegobin et al^8^ introduced a novel desensitization protocol starting at 150 mg twice daily, and they successfully escalated to the full dose of 600 mg twice daily over 23 days without rash recurrence. However, when Rodriguez et al^9^ adopted this same approach, their patient experienced a sore throat, erythematous rash, and low-grade fever within 1 day of reinitiating alectinib at 150 mg twice daily, highlighting the variable success of this method. Nagase et al^10^ described the utility of rapid desensitization therapy for alectinib, in which gradual dose escalation occurs over a single day. However, this method was unsuccessful in our second case, where rash recurred shortly after reintroduction despite undergoing rapid desensitization therapy, prompting dose reduction. Kimura et al^5^ were the only group to utilize concurrent systemic glucocorticoids during alectinib reintroduction. However, the steroid dose was relatively low—prednisolone 10 mg daily—and alectinib was restarted at just 20 mg twice daily, with titration reaching a maximum of 200 mg twice daily.^5^ This limited response suggests that low-dose steroids may be insufficient to support more reintroduction of alectinib at higher doses.

In contrast to prior reports, our single-center retrospective review demonstrates that concurrent use of a high-dose prednisone taper (≥40 mg) may facilitate reintroduction of alectinib at moderately reduced doses (150 mg daily-450 mg twice a day) without the need for low starting doses or prolonged titration (Table I). The American Academy of Allergy, Asthma, and Immunology supports systemic steroid use for benign cutaneous reactions when continuation of the offending drug is necessary.^11^ This strategy may reduce treatment interruptions and enable return to higher dosing compared with desensitization alone, helping maintain disease control in patients with limited options. To minimize systemic immunosuppression with corticosteroids, prompt reintroduction of alectinib is particularly desirable.

**Table I:** Summary of patient data with the highest initial dosage of systemic corticosteroids, duration of alectinib cessation, and alectinib dosage at initiation and maximum dose reached after rechallenge

Patient	1/M/44 yo	2/F/65 yo	3/F/49 yo
Alectinib cessation time	12 d	7 d	7 d
Starting steroid dose	80 mg	60 mg	40 mg
Alectinib dose at initiation	300 mg twice a day	150 mg daily	450 mg twice a day
Max dose of alectinib	450 mg twice a day	750 mg daily	450 mg twice a day
Time to max dose	∼3 wk	∼6 wk	
Overall duration of alectinib therapy (initiation to max dose)	7 mo (ongoing)	7 mo	4 mo
Clinical therapy response to alectinib	Partial response	Partial response	Partial response

*F*, Female; *M,* male; *yo*, years old.

Two of our cases successfully tolerated alectinib doses of 450 mg twice daily, whereas the third case was titrated to 750 mg daily without rash recurrence (Table I). Although these doses fall below the standard 600 mg twice daily regimen established in the ALEX trial, pharmacokinetic data suggest that not all patients require full-dose alectinib to achieve target drug exposure. In the J-ALEX trial, alectinib at 300 mg twice daily was established as the standard dosing regimen, demonstrating progression-free survival comparable to higher doses used elsewhere. This efficacy is thought to be influenced by lower average body weight and differences in drug metabolism in the Japanese population.^7^ Additionally, pooled exposure–response analyses of Western patients in ADAPT ALEC, a multicenter phase IV trial, demonstrated that 66% of individuals receiving alectinib 600 mg twice daily had plasma trough concentrations at or above 435 ng/mL, the therapeutic threshold established by Groenland et al.^12^^,^^13^ This highlights significant interpatient variability and raises the possibility that some patients may be receiving higher than necessary doses. Higher plasma concentration has not been associated with improved overall survival, supporting a plateau effect in clinical benefit and the feasibility of moderately reduced doses after adverse events.^12^

Notably, our cases also contribute histopathologic data that are absent from previous reports of alectinib-induced rash. Biopsies consistently showed findings characteristic of a morbilliform drug eruption. These features support the interpretation of the rash as a type IV delayed hypersensitivity reaction. There was no evidence of epidermal necrosis suggestive of Stevens–Johnson syndrome or toxic epidermal necrolysis, which would necessitate permanent discontinuation of the offending drug. The inclusion of biopsy data strengthens the clinicopathologic correlation and offers additional insight into the mechanism of this cutaneous toxicity.

Our case series is limited by its small sample size and the retrospective nature of case reporting. Additionally, the potential influence of prior immunotherapy exposure, present in at least one case, should be considered, as it may modulate the immune system and increase susceptibility to subsequent drug hypersensitivity reactions.^14^ Further studies are needed to determine the generalizability of this approach and to identify patient- or disease-specific factors that may predict efficacy of glucocorticoid-facilitated reintroduction.

In summary, we propose that a high-dose oral glucocorticoid taper combined with reintroduction of alectinib at a modestly reduced dose provides a practical alternative to previously published desensitization protocols. This approach may reduce treatment delays and lower the risk of rash recurrence, potentially enhancing outcomes for patients with *ALK*-rearranged non–small cell lung cancer.

## Conflicts of Interest

Dr. Waqar reports advisory board participation for Pfizer, Gilead, Janssen, Daiichi Sankyo, Boehringer Ingelheim, AstraZeneca, Amgen, and Adcendo. She also reports consulting for Gilead and AstraZeneca. Author Liao and Drs Daylan, Ward, and Ng have no conflicts of interest to declare.
